# Modeling Peripheral Olfactory Coding in *Drosophila* Larvae

**DOI:** 10.1371/journal.pone.0022996

**Published:** 2011-08-09

**Authors:** Derek J. Hoare, James Humble, Ding Jin, Niall Gilding, Rasmus Petersen, Matthew Cobb, Catherine McCrohan

**Affiliations:** Faculty of Life Sciences, The University of Manchester, Manchester, United Kingdom; University of Queensland, Australia

## Abstract

The *Drosophila* larva possesses just 21 unique and identifiable pairs of olfactory sensory neurons (OSNs), enabling investigation of the contribution of individual OSN classes to the peripheral olfactory code. We combined electrophysiological and computational modeling to explore the nature of the peripheral olfactory code *in situ*. We recorded firing responses of 19/21 OSNs to a panel of 19 odors. This was achieved by creating larvae expressing just one functioning class of odorant receptor, and hence OSN. Odor response profiles of each OSN class were highly specific and unique. However many OSN-odor pairs yielded variable responses, some of which were statistically indistinguishable from background activity. We used these electrophysiological data, incorporating both responses and spontaneous firing activity, to develop a Bayesian decoding model of olfactory processing. The model was able to accurately predict odor identity from raw OSN responses; prediction accuracy ranged from 12%–77% (mean for all odors 45.2%) but was always significantly above chance (5.6%). However, there was no correlation between prediction accuracy for a given odor and the strength of responses of wild-type larvae to the same odor in a behavioral assay. We also used the model to predict the ability of the code to discriminate between pairs of odors. Some of these predictions were supported in a behavioral discrimination (masking) assay but others were not. We conclude that our model of the peripheral code represents basic features of odor detection and discrimination, yielding insights into the information available to higher processing structures in the brain.

## Introduction

In the peripheral olfactory system, odors are represented by a combinatorial code comprising the responses of multiple classes of olfactory sensory neurons (OSNs). Investigation of the contribution of individual OSNs to this code is hampered by complexity; identification of specific OSNs *in situ* is difficult as most animals possess tens or thousands of cells of each OSN class. In contrast, the olfactory system of the *Drosophila* larva comprises 21 unique pairs of OSNs, most expressing just a single class of olfactory receptor (OR), and each projecting to its cognate glomerulus in the larval antennal lobe [1]–[3]. We can record the electrophysiological activity of individual OSNs *in vivo*, and the larva's genetic tractability enables analysis of the response profiles of individual, identifiable OSNs expressing specific ORs [4]. This system provides us with the possibility of describing the peripheral olfactory code for a complete OSN population in an intact organism.

In a previous study [4], we found that the firing responses of identified larval OSNs to specific pure odors were variable. OSNs of a given class responded reliably to some odors, but not to others. This variability was consistent for specific odor-OSN pairs and was not dependent on odor type or concentration, stimulus duration, genotype or inter-individual differences [4]. Decisively, in larvae expressing only two functional OSNs, one OSN class showed 100% responses to repeated, identical presentations of a given odor, whilst the other OSN class showed variable (<100%) responses to the same presentations of the same odor [4]. Thus, for some odor-OSN pairs, firing responses vary to the extent that they are sometimes statistically indistinguishable from background ‘noise’.

The response variability of individual OSNs implies that information reaching the CNS from individual OSNs may be ambiguous. We wished to explore how more reliable coding might emerge at the population level. To address this, we chose a Bayesian decoding approach [5] that would enable us to estimate how much odor identity information can be extracted from OSN activity by downstream neural circuits – in other words, how accurately a target odor can be identified based on the raw peripheral activity alone.

First, we exploited the ability to create larvae expressing just one functioning class of OSN to characterise the electrophysiological response profiles of 19 of the 21 OSNs to a panel of 19 biologically-relevant pure odors. This provided quantitative spike frequency information, including both reliable and variable responses. We then asked how the system could use this information to identify target odors. We used the recorded firing activity of identified OSNs during presentation of specific odors to develop a computational model, incorporating both unambiguous responses and ‘responses’ that were not statistically different from spontaneous activity. Finally we explored how effective the model was at discriminating between pairs of odors and tested its predictions using behavioral assays.

## Methods

### *Drosophila* strains

The *w^1118^* strain was obtained from the Bloomington stock centre. All other strains (*OrX*-Gal4, UAS-*Orco* and *Orco* ^−^) were gifts from Leslie Vosshall (Rockefeller University, NY). (Note that ‘*Orco*’ is the revised name for *Or83b*, and is applied to all orthologous insect genes [6].) Larvae with one functional pair of OSNs (‘single-functional-OSN’ lines) were created by making the appropriate [*OrX-*Gal4/UAS*-Orco; Orco^−/−^*] crosses. This procedure is effective because the ORCO protein is a co-factor required for correct expression of olfactory receptor proteins in *Drosophila*, so that *Orco^−/−^* larvae are anosmic [7]. Stocks were maintained at 25°C under a 12∶12 L∶D cycle and fed on standard oatmeal and molasses medium. We studied the individual responses of 20 OR classes of single-functional-OSN larvae: Or1a, Or13a, Or22c, Or24a, Or30a, Or33a, Or33b, Or35a, Or42a, Or42b, Or45a, Or45b, Or47a, Or49a, Or59a, Or63a, Or67b, Or74a, Or82a and Or83a. Or33b and Or 47a are expressed in the same OSN [7]; because the [*OrX-*Gal4/UAS*-Orco; Orco^−/−^*] line rescues Orco function in whichever cell the *OrX* driver is expressed, both Or33b and Or47a are rescued in the OSN when either the *Or33b-Gal4* or the *Or47a-Gal4* driver is expressed. As a result, we were able to study the responses of 19 of the 21 larval OSN classes. The 20 ORs (19 OSNs) tested here were the only ORs detected in larvae of this strain using [*OrX-*Gal4/UAS-GFP] in a previous study [7].

### *In vivo* electrophysiology

Our previously described method [4] was used. A third-instar larva was picked from its rearing tube and immobilized on a matchstick using parafilm. A glass microelectrode filled with *Drosophila* larval ringer [8] was inserted into the cuticle at the base of one of the paired dorsal organs (the sole sites of larval olfaction) on the head. An earth was put into contact with the larval body and a reference electrode inserted into the abdomen. Electrical activity was acquired using a Neurolog system (Digitimer Ltd., UK); an AC preamp subtracted reference electrode activity from the recording electrode activity, and the analog signal was amplified and filtered before being converted to a digital signal and analyzed offline using Spike2 software (Cambridge Electronic Design, UK; see [4]). Each recording contained the activity of a random sample of up to 8 of the 21 larval OSNs. We could therefore record a specific OSN in around 30% of recordings [4].

### Odorants

Nineteen biologically-relevant pure odorants were used: five alcohols (butanol, pentanol, hexanol, octanol and nonanol), six aliphatic esters (ethyl acetate, propyl acetate, butyl acetate, iso-amyl acetate, pentyl acetate and methyl caproate), two aromatics (benzyl acetate and anisole) two alkyl aldehydes (heptanal and octanal), one ketone (2-heptanone), one terpene (r-carvone) and two organic acids (hexanoic and nonanoic acid). All were from Sigma-Aldrich or BDH Laboratory Supplies and were of the highest purity available. Odorants were mixed to a final concentration of 2% with distilled water at room temperature (∼25°C) and shaken immediately before delivery to maintain an even concentration. During recording air was delivered continuously at a rate of 3 ml s^−1^ from a 2 mm-diameter pipette tip positioned 5 mm away from the head of the larva. The air was bubbled through distilled water in a sealed conical flask prior to its release. During stimulation, the air-stream was redirected for 1 s through a second conical flask containing the odor solution, using an electronically-controlled valve. Distilled water was used as a control in all experiments. There was no effect of either odor concentration or stimulus flow rate on the reliability of responses for a given odor-OSN combination (see Figure S1).

### Bayesian modeling

To determine how well the peripheral olfactory system can identify and discriminate the odors used in our study, we used a Bayesian decoding approach (reviewed in [5]). We wanted to know how accurately a given target odor (chosen from a set of N odors) could be identified (decoded) based on the spikes fired by the OSN population.

We denote the N = 19 odors by *o_i_* (i = 1…19). Let P(*o_i_*) denote the probability that the target odor on any given trial was odor *o_i_*. *r_j_* denotes the number of action potentials fired by the *j*th OSN (*j* = 1…15; all except Or45b and Or49a) in the time window of duration 1 s, starting at stimulus onset. P(r_j_|o_i_) denotes the conditional probability that the *j*th OSN fires *r_j_* spikes in response to delivery of odor *o_i_*. **r** = [*r_1_,r_2_,…,r_15_*] denotes the population response of all the OSNs on a given trial, and P(**r**|*o_i_*) the conditional probability that the population response **r** occurs in response to odor *o_i_*. Using Bayes' identity, knowledge about which odor was presented on a given trial can be gleaned from the neuronal responses:
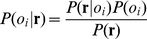
(1)Here P(*o_i_*|**r**) is the conditional probability that odor *i* was delivered, given that population response **r** was observed. 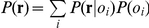 is the unconditional probability of the population response. Since in this study the OSNs were recorded individually, we approximated the conditional probability P(**r**|*o_i_*) as a product of marginals 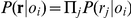.

We used a leave-one-out cross-validation approach. All trials except one (for each OSN-odor combination) were first used to fit the parameters of the model from the electrophysiological recordings, as detailed below. Then the remaining trial was used to test the performance of the model. To obtain reliable performance data, this training-test cycle was repeated 4000 times. All data on decoding errors reported in Results were obtained by this cross-validation procedure and are therefore ‘prediction errors’ (not ‘training errors’).We fitted the model parameters in the following way. We found that the probabilities P(*r_j_*|*o_i_*) could be accurately approximated by truncated Gaussian distributions. By combining data across all larvae with a given single functional OSN type, we had on average 8 trials (minimum 6) of the response of each OSN to delivery of each odor which induced a significant response, according to the criteria detailed in Results. We fitted the data for each of these OSN-odor combinations individually using a truncated Gaussian; the parameters of the truncated Gaussian were found by maximum likelihood. To be conservative, we assumed that responses of an OSN following stimulation with odors that were not ligands for that OSN were random and fitted such combinations to a truncated Gaussian, using spontaneous activity of the OSN (1 s period prior to delivery of each odor, as well as responses to non-ligand odors for that OSN).

The next step was to test the model's performance. We synthesized population responses **r** to a given target odor by, first, identifying which OSNs exhibited a significant response to the target odor and then, for each of these OSNs, randomly selecting one of its responses to that odor. For the other OSNs (those with no significant response to the target odor), one of the spontaneous ‘responses’ was selected. The maximum of the likelihoods P(*o_i_*|**r**) was selected as the ‘predicted odor’. If the predicted odor matched the target odor, this was counted as a correct decision, and *vice versa*. To reliably estimate the probability of a correct decision, the entire procedure was repeated 4000 times.

To determine chance levels of percent correct, we studied the performance of the model under the null hypothesis that the relationship between odors and responses was random. We randomized the relationship between responses and which odors had actually elicited them, refitted the parameters of the model, and retested it as described above. By repeating this procedure 4000 times, we determined an upper (*p* = 0.01) confidence level (6.96%) above which predictions could be considered to be statistically significant.

### Behavior

We used a standard locomotor assay to quantify the response of larvae to individual odors. Briefly, at least 20 third instar larvae were placed at the centre of a 9 cm diameter Petri dish that was covered with 2.5% agar. 2.5 µl of an undiluted odor source was loaded onto a small circle of filter paper placed on the lid of an Eppendorf tube situated on one side of the dish. The dish was placed on a grid that divided the plate into two halves, with a 10-mm diameter central start zone. The lid of the dish was replaced and after 5 minutes the distribution of the larvae in the three zones (attracted, repulsed, not responding) was noted. A behavioral response index ((n_att_−n_rep_/n_tot_)×100) was then calculated [10]. To estimate how well larvae discriminate between two odors, we carried out a masking experiment [9], in which the behavioral locomotory response to a point source of odor A was tested in the absence and presence of a background of odor B. The latter was generated by evenly spacing 5×1 µl aliquots of odor B on a 9 cm filter paper, which rested between the Petri-dish and the lid. Odor A was placed on an Eppendorf lid on one side of the dish as described above.

## Results

### Spontaneous and odor-evoked firing activity in identified OSNs

We used 20 single-functional-OSN lines to investigate spontaneous (background) activity and responses to odors in identified OSNs. Reliably identifying the activity of a rescued OSN in these lines was straightforward when there was a response to an odor, as only one OSN showed altered activity. The OSN could then be simply tracked through the same recording on the basis of its unique action potential amplitude and shape [4]. We never detected more than one responding OSN within a given recording and therefore concluded that in every case this was the rescued OSN.

In the absence of odor stimulation OSN classes showed different and varying levels of spontaneous activity (Table 1). The mean spontaneous activity of rescued OSNs varied from 0.7±0.2 Hz (Or33b/47a) to 7.9±1.6 Hz (Or83a) with an overall range of 0–27 Hz; these values encompass the range of spontaneous activity for OSNs from non-genetically manipulated *w^1118^* larvae (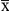 = 7.6±0.62 Hz; n = 296, range = 0–31; [4]), and of unresponsive OSNs in *Orco^−/−^* larvae (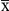 = 3.63±0.5 Hz; n = 15, range = 1–15).

**Table 1:** Summary of electrophysiological activity of single olfactory sensory neurons (OSNs) in *Drosophila* larvae.

Spontaneous activity (Hz)						Firing rate during stimulation (Hz)						
						*Response above criterion*					*Absolute*	
OSN	Mean	SE	n	Min.	Max.	Mean	SE	n	Min.	Max.	Min.	Max.
Or1a	3.2	0.8	24	0	16	4	0.8	30	0	21	0	37
Or13a	1.5	0.2	24	0	4	7.9	2.1	18	0	30	0	59
Or24a	7.3	0.8	24	0	22	9.6	1.1	46	−9	38	0	51
Or30a	3.8	0.3	24	0	6	11.1	1.4	10	0	67	0	74
Or33b/47a	0.7	0.2	24	0	3	10	0.5	8	3	41	2	50
Or35a	7	0.9	24	0	20	16.4	1.4	16	−32	109	0	115
Or42a	4	0.8	24	0	17	41.6	1	38	0	133	0	137
Or42b	5.5	1.1	24	0	18	14.8	1.4	12	0	80	0	104
Or45a	2.7	0.8	24	0	8	6.1	0.5	22	0	19	0	28
Or45b	1.9	0.4	24	0	6	23.5	1.4	8	4	45	9	53
Or49a	5.2	0.9	24	0	18	−3.2	0.4	21	−15	0	0	10
Or59a	1.2	0.3	24	0	4	17.6	1	17	0	61	0	69
Or63a	1.2	0.3	24	0	5	15.9	1	14	0	43	0	49
Or67b	6	0.4	24	0	15	8.1	0.4	36	0	44	1	48
Or74a	4.9	0.5	24	0	12	15.1	0.9	22	−8	51	0	60
Or83a	7.9	1.6	24	0	27	14.8	0.8	14	0	33	3	47

Spontaneous activity = the activity of a single OSN in the second prior to each olfactory stimulation. Firing rate = the activity of a single OSN during 1 second stimulation with one of 19 odors. Min. and max. denote the minimum and maximum rates observed. Response above criterion = change in spike frequency above/below the probabilistic response criterion (a change of ±5 Hz during stimulation as compared to the spontaneous activity seen in that OSN in the 10 s prior to stimulation – see Materials and Methods). Absolute = absolute OSN activity during stimulation. Larvae with a single functional OSN were [OrX-Gal4/UAS-Orco ; Orco−/−], constructed following the protocol in [7]. Or33b and Or47a are co-expressed in the same neuron, so their data were pooled. To be certain that spontaneous activity was obtained from a functional OSN, a response to at least one odor had to be detected in that OSN. No responses were detected for Or22c, Or33a and Or82a, so there are no data for these three classes of OSN.

**Figure 1:**
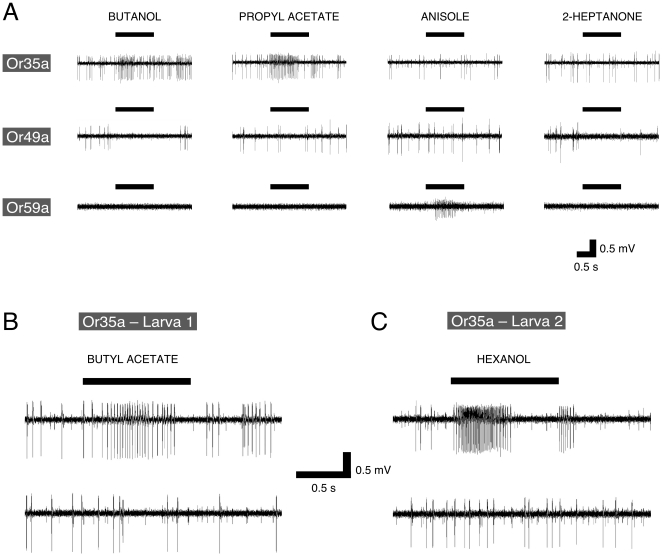
Electrophysiological activity of single, identified OSNs during 1 s stimulation (bar) with odors. Recordings are from single-functional-OSN larvae. **A.** Each trace for a given OR class is from the same larva. The Or35a OSN was activated by butanol and propyl acetate, but showed no response to anisole or 2-heptanone; the Or49a OSN was inhibited by butanol and 2-heptanone, but showed no response to the other two odors; the Or59a OSN was activated by anisole, but showed no response to the other three odors. **B, C.** The Or35a OSN showed variable responses to both butyl acetate and hexanol. B and C were recorded from separate larvae, each of which showed a response (top trace) or no response (lower trace) to identical presentations of an odor.

Nineteen pure odors were each tested on at least 60 OSNs from each of the 20 single-functional-OSN lines. Once a ligand had been identified for a particular OSN class, we were able to know if the rescued OSN was present in any particular recording. For three OSN classes – Or22c, Or33a and Or82a – we were unable to identify any ligands using electrophysiology. Odors were delivered as 1s pulses, in a random order, with a 2 min inter-stimulus interval. Specific odor-OSN combinations that gave a response were presented at least eight times. Typical electrophysiological recordings for three OSN classes and four odors are shown in Figure 1A; the traces for each OSN class were from a single larva and also included spontaneous activity in other, non-functional OSNs. There was no evidence that the activity of a responding OSN altered the spontaneous activity of the non-functional *Orco^−/−^* OSNs, confirming our previous report [4]. There were no significant correlations between the maximum absolute activity observed for each OSN class during odor stimulation and the maximum level of spontaneous activity (*r_15_* = −.052, *p* = 0.847) (Table 1), between maximum absolute activity during odor stimulation and mean spontaneous activity (*r_15_* = 0.024, *p* = 0.929), or between maximum activity during a response and mean spontaneous activity (*r_15_* = 0.006, *p* = 0.983), indicating that response intensity in a given OSN was independent of its level of spontaneous activity.

**Figure 2:**
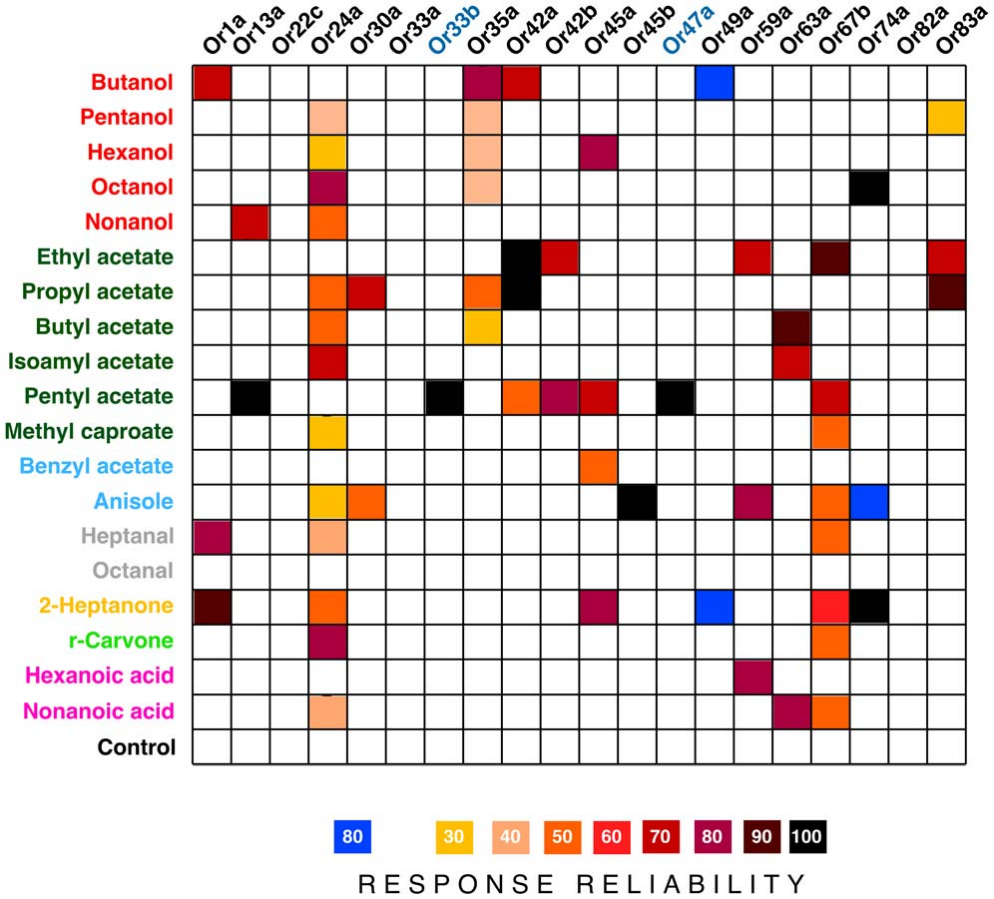
Summary of electrophysiological responses of identified OSNs to a panel of 19 odors. Blue squares indicate inhibition; red to brown show excitation. Numbers in ‘response reliability’ key indicate the percentage of times that an identified OSN responded to a given odor, rounded up to the nearest 10%. OR33b and OR47a are co-expressed in the same OSN (indicated in blue) and show identical response profiles.

For some odor-OSN combinations, responses were highly variable (Figure 1B,C). In the light of this, together with variations in spontaneous activity, it was important to provide a criterion to define a significant response. We used our previously-described, probabilistic ‘response criterion’ for which the 0.05 probability of making a Type 1 error in describing the activity of a particular OSN as a ‘response’ corresponds to a change in firing rate of ±5 Hz during stimulation as compared to the extremes of spontaneous activity seen in that OSN in each of the 10 s prior to stimulation [4]. This objective, probabilistic definition, based on the known activity of a particular OSN, is preferable to an arbitrary threshold, or to no criterion at all. For each odor-OSN pair we calculated the percentage of responses to a given odor that exceeded the criterion, i.e. those for which the change in firing rate was statistically distinguishable from noise. The data are summarized in Figure 2 (and presented in full in supplementary Figures S2 and S3, which also include the mean response intensity – spike count – above criterion).

60/361 (16.6%) of the odor-OSN combinations studied produced a response. As expected, the same response profile was generated by the rescue of *Or33b* and of *Or47a*, which are co-expressed in the same OSN [7]. Both these lines were activated only by pentyl acetate, and did not share this profile with any other single-functional OSN line. No two OSN profiles were the same, confirming that each of the larval OSN classes is unique, and that our data represent the responses of 19 of the 21 larval OSNs. Octanal did not activate any OSN, while pentyl acetate caused excitation in seven OSN classes. Some OSN classes responded to no odor (n = 3), or up to 13 odors (n = 1), but most showed considerable selectivity in their response profile; 11/19 OSN classes responded to just 1–3 odors.

Using our stimulus regime, the majority of odor-OSN response profiles were variable (i.e. responses were above criterion on <100% of trials, Figure 2), and most (57/60) were excitatory. Only three odor-OSN combinations produced inhibition; the Or49a OSN was inhibited by butanol and 2-heptanone (Figure 1A), while the Or67b OSN was inhibited by anisole. The remaining 301 odor-OSN combinations consistently yielded no response. There was no significant correlation between mean response intensity (spikes/s) and the frequency with which a response above criterion was observed for each odor/OSN combination (*r_56_* = .245, *p* = 0.066).

### A Bayesian decoding model of the peripheral code

**Figure 3:**
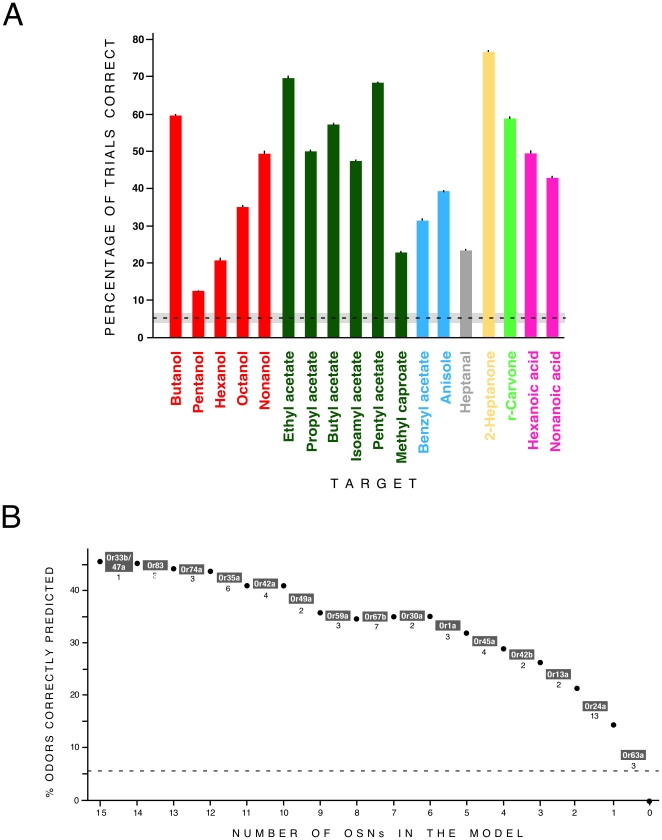
Decoding results of the Bayesian model of peripheral processing. **A.** The model was presented with responses to each of the 18 odors (‘target’) and had to identify which odor induced the response profile. The graph shows the mean percentage of trials (± SEM) on which the model correctly identified the target, using 4000 simulations for each odor. Dashed line indicates the percentage expected by chance, grey band corresponds to *P*<0.01 confidence limits (see Methods). **B.** Relative contribution of each OSN class to the accuracy of the model. Data show mean accuracy levels with a progressive reduction in the number of OSN classes in the model. Standard errors are smaller than the size of the data points. OSN classes contributing least to the ability of the model to accurately predict odors were iteratively removed. The number of odors detected by the removed OSN class is given underneath each OSN label. Dashed line indicates chance level of correct odor prediction. The X-axis shows the number of OSNs in the model, with the full model (n = 15) as the first value.

Our consistent finding of considerable variation in responses of OSNs to specific odors raised the question of whether the robustness of odor discrimination is increased by integration of information at the population level. We hypothesized that reliable information transmission emerges at the ensemble level by integrating the responses of multiple OSNs. To test this hypothesis, we constructed a Bayesian decoding model that integrates the responses of multiple OSNs in a statistically efficient manner. We used our electrophysiological data from single-functional-OSN larvae to develop the model (see Methods). We included the actual spike count for each functional OSN during 1 s stimulation with each odor, regardless of the level of spontaneous activity. Thus the model incorporated responses as well as spontaneous (background) activity of both responding and non-responding OSNs, which all together contribute to the combinatorial code for a given odor. Four OSNs were excluded due to shortage of (Or45b), or lack of (Or22c, Or33a and Or82a) electrophysiological data. One odor (octanal) was excluded because no single-functional-OSN strain showed an electrophysiological response to it. The input to the model on any given trial therefore consisted of randomly selected samples of the activity of each of 15 OSNs during presentation of one of 18 target odors. The corresponding output of the model was a prediction of the odor most likely to have elicited the input – the model was effectively required to identify the input as one of the target odors. The fact that the OSN responses exhibit considerable variability in their response to a given odor, makes this a demanding 18-alternative forced choice task; chance level was 5.6%. The model's performance for each of the 18 target odors is plotted in Figure 3A. Despite the considerable variability of the firing responses for most odor-OSN combinations, every target was significantly correctly predicted. The mean accuracy of stimulus identification across all odors was 45.2±0.2%, eight times greater than chance. However, not all odors were equally well predicted. The most accurate prediction level was for 2-heptanone (76.8±0.3% of trials); the least accurate was for pentanol (12.3±0.2%). The model was particularly efficient at detecting aliphatic esters (ethyl…pentyl acetate) (range = 49.1±0.4% to 69.4±0.3%).

To determine the robustness of the peripheral odor representation, we examined the effect of progressively eliminating individual OSNs. At each stage we recomputed the prediction accuracy based on all 15 subsets of 14 cells and determined the OSN whose removal produced least performance decrement. This OSN was then eliminated. This procedure was repeated until only a single OSN remained (Figure 3B). The first OSN to be removed was Or33b/47a, which had virtually no effect on the accuracy of the model (following removal of this OSN, the model's accuracy actually increased from 42.23±0.08% to 45.22±0.09%), suggesting that information from this OSN is not necessary for the detection of the odors studied here (this OSN responded only to pentyl acetate, which was detected by five other OSNs). Indeed, the first five OSNs (Or33b/47a, Or83a, Or74a, Or35a and Or42a) were removed from the model with only a slight decline in its accuracy (from 42.23±0.08% to 40.84±0.08%). There was no significant correlation between the order in which OSNs were removed from the model and number of odors to which they responded (*r_14_* = 0.362, *p* = 0.204), showing that the efficiency of the model is not just based on the number of odors that each OSN can detect. Not surprisingly, as OSNs were removed from the model, certain odors could no longer be predicted at all. For example, when only Or63a was left, the overall (mean) accuracy of the model was 14.73±0.03%, but this OSN was unable to correctly identify some odors, such as butanol….nonanol.

### Testing the model using behavioral assays

**Figure 4:**
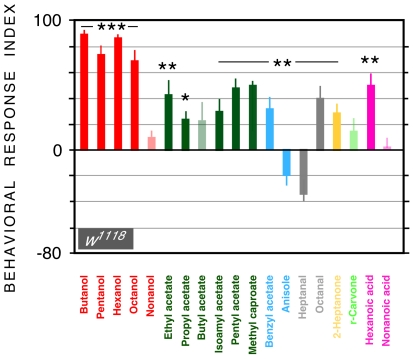
Behavioral responses of wild-type *w^1118^* larvae to 19 odors. Mean behavioral response indices ± SEM. Larvae were stimulated with a point source of odor in a mass behavioral test. Response indices were compared with a theoretical value of zero using one-sample t-tests. * = *P*≤.05; ** = *P*≤.01; ** = *P*≤.001. n = 8 assays per odor.

Although the model was based on data collected from 19 OSNs, rather than the full complement of 21, and despite obvious differences in stimulus duration (1 s for electrophysiology vs 5 min for behavior), we decided to explore whether the model could nevertheless make useful predictions about behavioral responses to odors. We tested responses of wild-type, 21-functional OSN *w^1118^* larvae to individual odors using a mass locomotory assay. The results are shown in Figure 4. A significant response index – either attraction or repulsion - was obtained for 15 out of 19 odors. To explore any relationship between the model's accuracy in predicting a target odor (Figure 3A) and the strength of the behavioral response (response index, either positive or negative) to that odor, we carried out a correlation analysis (octanal was omitted, since this was not used to generate the model). There was no correlation between the two parameters. Some odors that were predicted well by the model yielded a weak behavioral response (e.g. propyl acetate, 2-heptanone, ), and vice versa (e.g. pentanol, hexanol).

**Figure 5:**
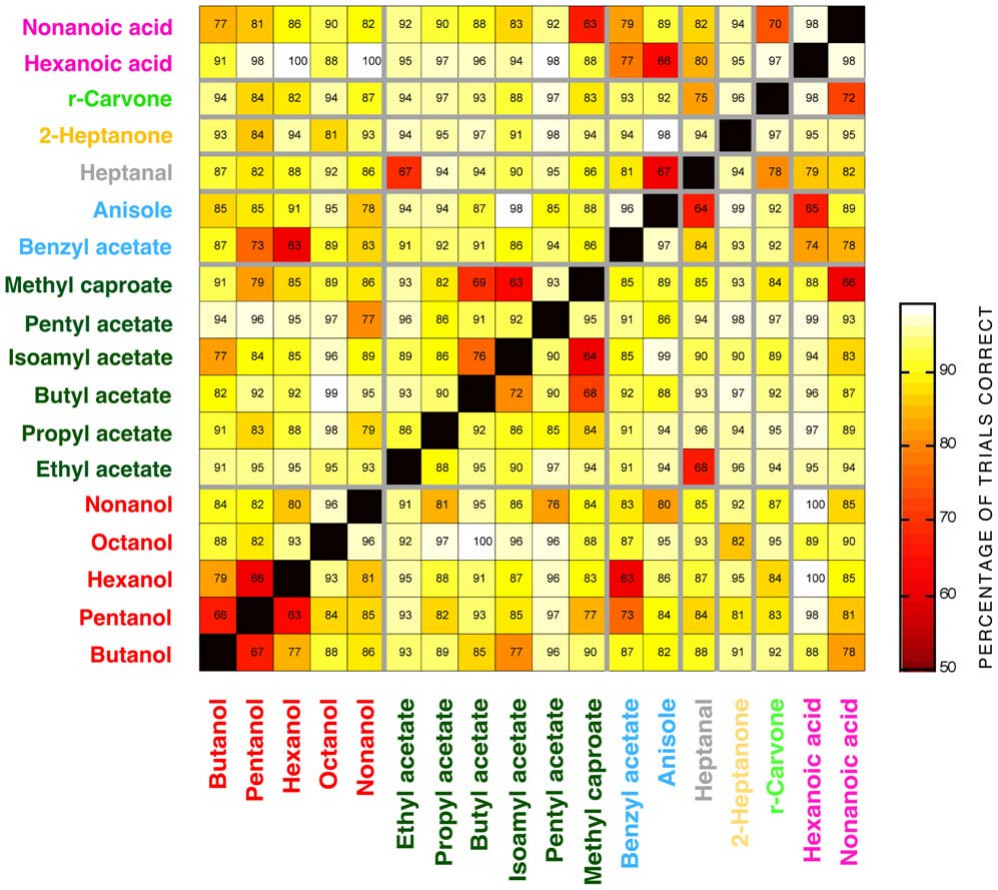
Ability of the Bayesian model to discriminate between pairs of odors. The model was presented with OSN responses to each pair of odors and required to discriminate between them. The table shows the % of times an odor pair was correctly discriminated; each discrimination was run through the model 4000 times. Values are not strictly reciprocal on either side of the diagonal because of the sampling method used by the model. Chance discrimination = 50%.

To further explore the ability of the model to reflect behavior, we examined how well a Bayesian model could discriminate between pairs of odors: that is, when the model's target was odor A, how often the OSN activity profile of that odor could be distinguished from that of odor B. For each odor pair of interest, we constructed a Bayesian model based on the 15 OSNs identified above and trained it to discriminate between the two odors. (The procedures were otherwise identical to those used above). Figure 5 presents a discrimination matrix showing the ability of the model to discriminate all possible odor pairs. The model was presented with OSN responses to each pair of odors and required to discriminate between them. Chance performance was 50%. Every odor pair was discriminated above this level and, with 11 exceptions, showed a discrimination value of ≥75%. The lowest levels of discrimination were generally found between structurally similar pairs of odors (e.g. pentanol/hexanol – 63–66%). Within functional groups, the highest levels of discriminability were detected between hexanoic and nonanoic acid (98%), while the most consistent discriminability was seen within the four homologous aliphatic esters (ethyl… pentyl acetate; 86–96%).

**Figure 6:**
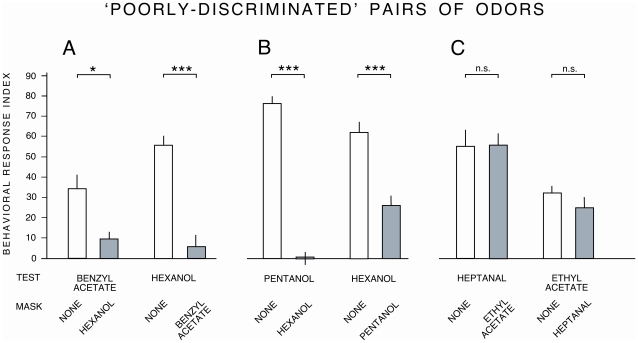
Testing the Bayesian model using behavioral discrimination of odor pairs. Masking experiment for pairs of odors predicted by the model to be poorly discriminated (63–68% discriminability). Control *w^1118^* larvae were tested in a mass olfactory experiment, presented with a localised odor (‘test’) and a masking odor (‘mask’). For full details, see text. * = *P*<0.01, *** = *P*<0.001, n.s. = not significant. n = 8 assays per condition.

**Figure 7:**
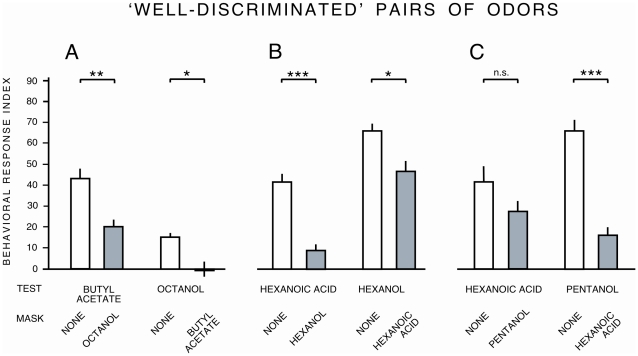
Testing the Bayesian model using behavioral discrimination of odor pairs. Masking experiment for pairs of odors predicted by the model to be well discriminated (98–100% discriminability). Control *w^1118^* larvae were tested in a mass olfactory experiment, presented with a localised odor (‘test’) and a masking odor (‘mask’). For full details, see text. * = *P*<0.01, *** = *P*<0.001, n.s. = not significant. n = 8 assays per condition.

We tested the output of the discrimination matrix by studying the behavior of wild-type larvae. We used a ‘masking test’ [9], in which wild-type larvae were required to detect a test odor in the presence of a continuous background of the other, masking, odor. We chose pairs of odors that were either poorly discriminated by the model (benzyl acetate and hexanol; pentanol and hexanol; ethyl acetate and heptanal – 63–68% discriminability; Figure 6A–C) or that were well discriminated (butyl acetate and octanol; hexanoic acid and hexanol; hexanoic acid and pentanol – 98–100% discriminability; Figure 7A–C). If two odors are hard to distinguish, larvae should find it difficult to detect the test odor against the background masking odor; the task should be easier for odor pairs that are easy to distinguish.

In some cases the model was apparently a good predictor of behavioral discrimination. For example, two of the three odor pairs that were predicted by the model to be poorly discriminated - benzyl acetate and hexanol, and pentanol and hexanol - were also poorly discriminated in the masking test (Figure 6A,B). However, for the heptanal/ethyl acetate pairing, responses to each odor were not significantly reduced in the presence of the other as a mask, demonstrating good behavioral discrimination (Figure 6C). Behavioral response indices to butyl acetate and octanol, and to hexanol and hexanoic acid were all significantly reduced in the presence of the other odor as a mask (Figure 7A,B), despite the model's prediction of good discrimination between these odor pairs. The third pair of odors, predicted to be well discriminated, yielded asymmetrical data in the masking test. The response to hexanoic acid was not significantly reduced in the presence of a pentanol mask, whereas in the reciprocal test the response to pentanol was significantly reduced in the presence of hexanoic acid (Figure 7C). These data show that predictions arising from our model of peripheral ONS coding are not always correlated with behavioral odor discrimination, and that discrimination as measured by the masking test is not reciprocal for every odor pair.

## Discussion

In this study we present the first description of peripheral olfactory coding in a near-complete (19/21) population of OSNs, and in an intact organism. We extend our previous finding that many odor-OSN combinations yield highly variable responses; based on an objective probabilistic criterion, many ‘responses’ are not statistically different from spontaneous changes in background firing activity in the same neuron [4]. Response uncertainty in the peripheral olfactory system has been reported for other organisms. Mouse MOR71 cells responded consistently to acetophenone but showed qualitative response variability to benzaldehyde [11]; similar differences have been reported in MOR23 cells [12]. In *Anopheles* mosquitoes, TE1A OSNs respond >80% of the time to 4-ethylphenol, but <20% of the time to pentanoic acid [13]. In the vast majority of organisms, where there are many neurons within each OSN class, quantitative and qualitative variability in responses provides a continuum of overall response intensity within the class. However, the *Drosophila* larva has only a single pair of OSNs in each class, so must‘cope’ with variability as an integral part of the peripheral code.

In apparent contrast to our findings, Asahina et al. [14] recorded odor-evoked calcium signals in OSN axon terminals within the larval antennal lobe and found that responses were predictable (invariant) for a given odor-OSN combination. However, axonal calcium imaging does not provide a complete reflection of firing activity in sensory dendrites. For example, the adult *Drosophila* antennal lobe shows presynaptic peptidergic suppression of calcium signals [15]. It is possible that similar presynaptic modulation also occurs in the larval brain, providing an initial step towards ‘sharpening up’ an apparently unreliable primary code.

Our study was an *in vivo* and *in situ* investigation of larval OSN function. Kreher et al. [9], [16] used a *Drosophila* ectopic expression system (the ‘empty neuron’ – larval ORs are expressed in adult OSNs in which normal adult OR expression is prevented) to study the ligand specificity of larval ORs. These two experimental approaches used different odor delivery methods, response criteria, life-stages, background strains of *Drosophila*, cellular contexts and odor/receptor combinations. Nevertheless, for the 14 ORs and eight odors shared by our studies there was a large degree of agreement. More than 70% of the findings of the two studies were the same in terms of the odors that did/did not induce a response in a particular OSN class. Of the remaining odor-OR combinations that produced a response in one study but not the other, most (68%) were cases where Kreher et al. [9] reported a response and we did not. These authors reported more instances of inhibitory responses than we found; this is partly due to their response criterion (using a different criterion, they described fewer examples of inhibition in their earlier report [16]). Differences in neuronal context might also account for differences in the nature of responses recorded.

Guo and Kim [17] modelled the function of *Drosophila* OR molecules by comparing electrophysiological data from the empty neuron preparation [18] and the protein sequence data of each class of OR. This investigation suggested that *Drosophila* ORs contain a pocket into which odor molecules bind and provided some insight into specific binding sites, in particular for unbranched primary alcohols (methanol… octanol) and the inhibitory response of the adult receptor Or47b. Combining our unique *in situ* data set for larval ORs and our model of OSN activity with this approach [17] might shed further light on OR and OSN function, in particular the differences observed between ORs expressed at either and both life-stages of this insect.

Our Bayesian modeling approach explored how accurate odor discrimination might emerge from an ensemble code incorporating variable responses. Both these responses and the unmodulated, spontaneous activity of non-responding OSNs must together form the overall code. It was therefore important for our model to incorporate all firing activity that occurred during presentation of a given odor, whether in responding or non-responding OSNs. This ensured that activity of the whole OSN population was taken into account when predicting target odors. Despite the variability in both levels of spontaneous activity and responses of OSNs, the model was able (on average eight times better than chance) to identify all of the odors tested from the raw electrophysiological data. These results are conservative, since they include information only about the number of spikes fired in a one second time window. Normally the brain would have access to a much richer sample of peripheral activity than this, including the temporal pattern of spikes, and over longer stimulation periods. Taking this into account, the model was surprisingly good. It also confirmed that the peripheral code is distributed; loss of activity from one or a few OSNs only slightly reduced its ability to accurately predict odors. The model did not identify all odors with equal accuracy. The most reliably identified were the ecologically significant homologous aliphatic esters (ethyl… pentyl acetate), encouraging us to think that the model does indeed reflect important aspects of sensory processing in this organism.

In creating the model, our aim was to generate a representation of the peripheral code. However, it was still interesting to explore its potential to predict behavior. There was no correlation between the model's ability to identify a target odor and the behavioral response index for the same odor. Similarly, when we tested discrimination between pairs of odors, the model was not a reliable predictor for behavioral discrimination. These findings were not surprising, and may have a number of explanations. First, the model may not adequately reflect the peripheral code owing to the limitations in our data set referred to earlier. In particular, we were able to record from only 19 of the 21 larval OSNs. The two ‘missing’ OSNs were present in the 21-functional OSN larvae used in the masking test, and may be decisive for accurate identification and discrimination of some or all of these odors. Second, the odor presentation regimes (timing, concentration) differed between electrophysiological and behavioral tests and this would be expected to influence the measured output. Third, behavioral output reflects the integrative processing of olfactory information by the brain, whereas the model was based on peripheral activity alone. The ability to detect and correctly identify a given odor is necessary, but not sufficient, to elicit a behavioral response to that odor. The latter also depends on the adaptive and behavioral relevance of the odor and may present as either attraction, repulsion, or no behavioral response. Brain processing could also explain how an odor pair that is poorly discriminated by the peripheral model (for example, heptanal/ethyl acetate) is much better discriminated by the whole animal; in this case, discrimination must be sharpened up centrally. In the adult fly brain, odor detection is sharpened by differential amplification and modulation of signals from OSNs and their cognate glomerulus, together with fast and rapidly accommodating firing responses in projection neurons [19]. Lateral inhibition between glomeruli, which sharpens the signal by increasing the signal∶noise ratio, appears to be particularly important [20]; intraglomerular inhibition may also play a role [21]. There are similar structures in the larval antennal lobe [22], and the output of OSNs and glomeruli is modulated by inhibitory local interneurons and projection neurons, at least one of which mediates concentration-invariant odor perception [14]. Such central processing could be used not only to enhance detection of individual odors but also to improve discrimination between odors. In the mammalian olfactory bulb, enhanced cholinergic neurotransmission both sharpens the olfactory receptive fields of mitral cells and increases behavioral pairwise odor discrimination [23]. A further observation from the behavioral masking tests was that reciprocal odor discrimination could be asymmetrical. In this test, the two odors are presented differently – one as a background odor and the other a localised source. The effects of these two kinds of presentation on odor gradients within the plate could influence the way in which each is perceived by the brain.

We conclude that, even if the model were deemed to be a fairly good representation of the basic peripheral code, assuming that its output is directly translating into odor-induced behavior implies that important and essential aspects of central processing will be overlooked. However, the model was able to identify a range of ecologically relevant odors on the basis of the peripheral responses they induce, supporting the view that the peripheral code can perform this task, albeit crudely, without the need for central processing. An interesting aim for future research will be to explore further how far the initial olfactory code embodied by our Bayesian model places constraints on olfactory behaviour, thus providing insight into the nature and function of central processing.

## Supporting Information

Figure S1 **Variable responses for a given odor-OSN combination are not a function of stimulus flow rate or concentration.** Individual *w^1118^* larvae (1–4) were stimulated with 2% butanol at three different flow rates (**A**) or 0.2% butanol at 30 ml/s (**B**). In all cases, butanol induced qualitative response variability; sometimes the OSNs responded, sometimes they did not. For clarity, responses are ordered in terms of whether there was a response or not; there was no order effect; stimuli were presented in random order.
(TIF)

Figure S2 **Electrophysiological responses of identified larval OSNs.** Larvae from nine single Or strains (Or1a – Or45a) were stimulated with 19 odors. Responses are given as mean (± SEM) firing rates of single OSNs above an objective response criterion. Percentages indicate the proportion of odor presentations that elicited a response above criterion when stimulated with a given odor, in preparations in which the functional OSN had been identified by showing a response to another odor (there are no percentages for the Or33b OSN, which responded to only a single odor). n≥8 tests per odor/OSN combination. (Data for Or13a, Or42a and Or42b are taken from [4], Figure 5).
(TIF)

Figure S3 **Electrophysiological responses of identified larval OSNs.** Larvae from eight single Or strains (Or45b – Or83a) were stimulated with 19 odors. Responses are given as mean (± SEM) firing rates of single OSNs above an objective response criterion. Percentages indicate the proportion of odor presentations that elicited a response above criterion when stimulated with a given odor, in preparations in which the functional OSN had been identified by showing a response to another odor (there are no percentages for the Or45b and Or47a OSNs, which responded to only a single odor). n≥8 tests per odor/OSN combination.
(TIF)
