# Supplementary figures and images for: Modeling Peripheral Olfactory Coding in Drosophila Larvae
Source: PLoS One. 2011 Aug 9;6(8):e22996. doi: 10.1371/journal.pone.0022996 (PMC3153476; doi:10.1371/journal.pone.0022996)

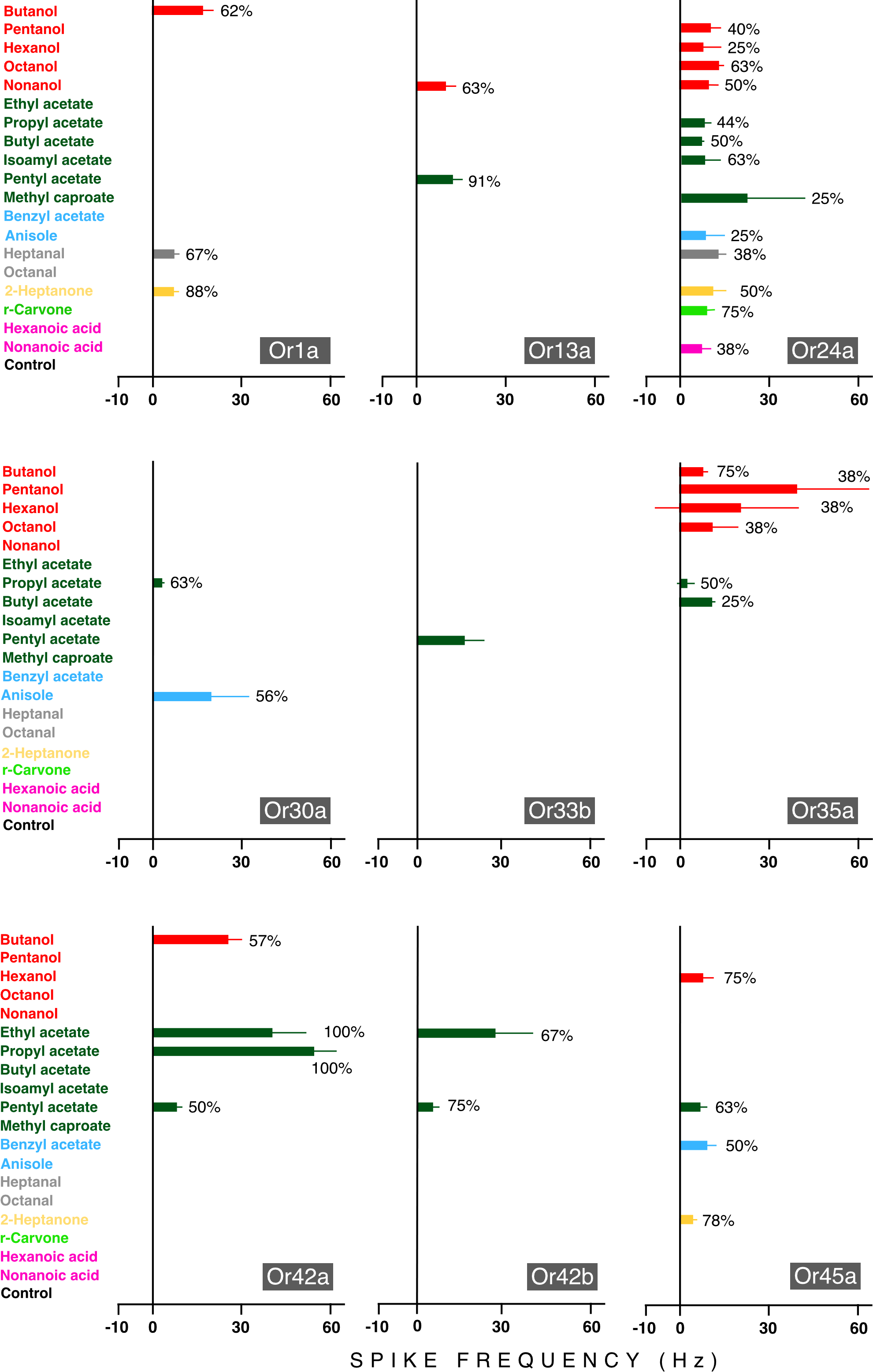

Supplement: Figure S2 — Electrophysiological responses of identified larval OSNs. Larvae from nine single Or strains (Or1a – Or45a) were stimulated with 19 odors. Responses are given as mean (± SEM) firing rates of single OSNs above an objective response criterion. Percentages indicate the proportion of odor presentations that elicited a response above criterion when stimulated with a given odor, in preparations in which the functional OSN had been identified by showing a response to another odor (there are no percentages for the Or33b OSN, which responded to only a single odor). n≥8 tests per odor/OSN combination. (Data for Or13a, Or42a and Or42b are taken from [4], Figure 5). (TIF) [file pone.0022996.s002.tif]

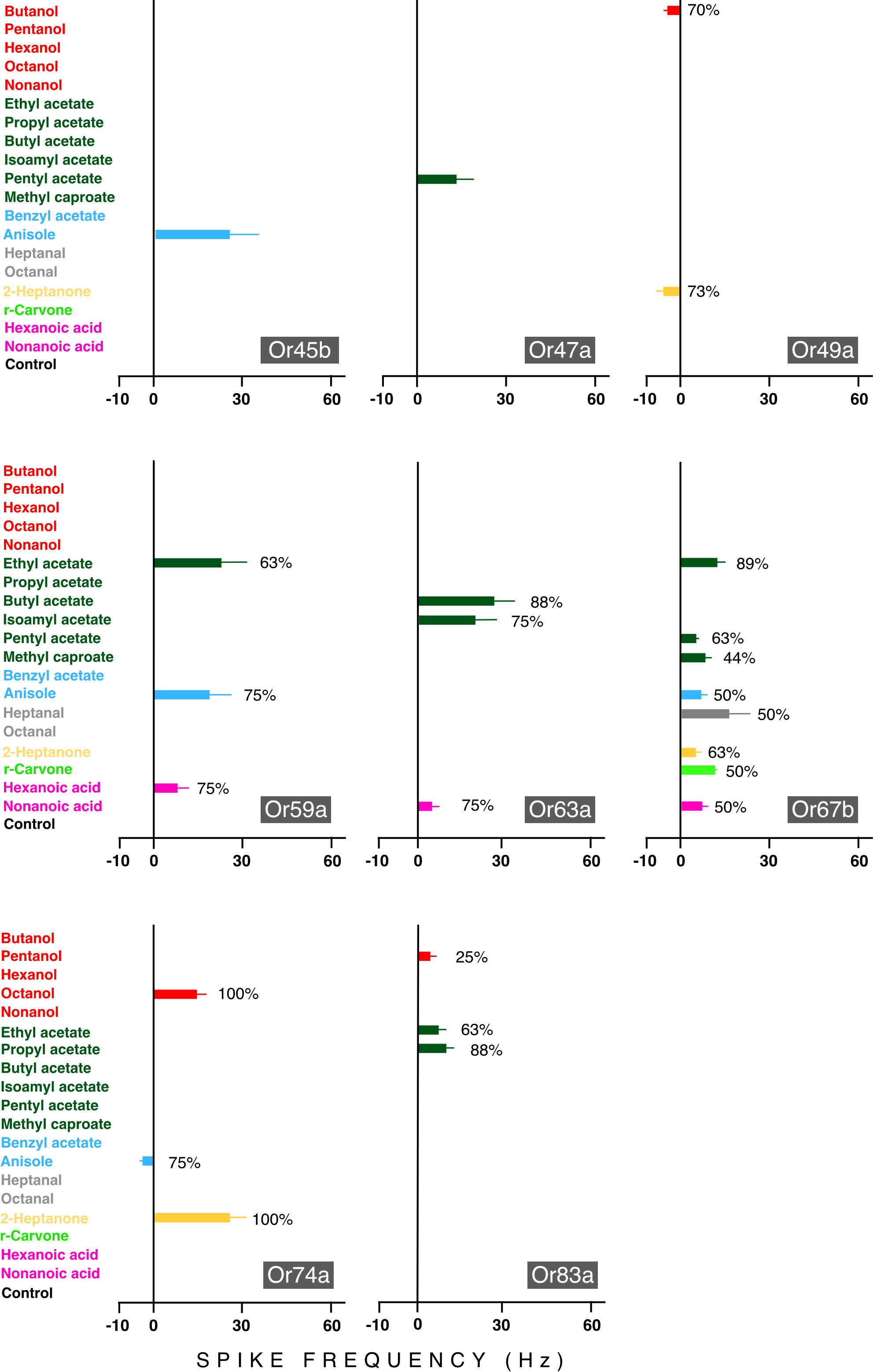

Supplement: Figure S3 — Electrophysiological responses of identified larval OSNs. Larvae from eight single Or strains (Or45b – Or83a) were stimulated with 19 odors. Responses are given as mean (± SEM) firing rates of single OSNs above an objective response criterion. Percentages indicate the proportion of odor presentations that elicited a response above criterion when stimulated with a given odor, in preparations in which the functional OSN had been identified by showing a response to another odor (there are no percentages for the Or45b and Or47a OSNs, which responded to only a single odor). n≥8 tests per odor/OSN combination. (TIF) [file pone.0022996.s003.tif]
